# The CXCR4-CXCL12 axis in Ewing sarcoma: promotion of tumor growth rather than metastatic disease

**DOI:** 10.1186/2045-3329-2-24

**Published:** 2012-12-18

**Authors:** Dagmar Berghuis, Marco W Schilham, Susy J Santos, Suvi Savola, Helen J Knowles, Uta Dirksen, Karl-Ludwig Schaefer, Jukka Vakkila, Pancras CW Hogendoorn, Arjan C Lankester

**Affiliations:** 1 Department of Pathology, Leiden University Medical Center, Leiden, The Netherlands; 2 Department of Pediatrics, Leiden University Medical Center, Albinusdreef 2, 2300, RC, Leiden, the Netherlands; 3 Dept. of Pathology, Haartman Institute and HUSLAB, University of Helsinki and Helsinki University Central Hospital, Helsinki, Finland; 4 Botnar Research Center, Nuffield Orthopedic Center, University of Oxford, Oxford, United Kingdom; 5 Dept. of Pediatric Hematology and Oncology, University Hospital Muenster, Münster, Germany; 6 Institute of Pathology, Heinrich-Heine University, Düsseldorf, Germany

**Keywords:** Ewing sarcoma, CXCR4, CXCL12 (stromal-cell derived factor-1 (SDF-1)), Chemokine, Growth signaling, Hypoxia, Metastasis, Prognosis, Therapy

## Abstract

**Background:**

Chemokine receptor CXCR4, together with its ligand CXCL12, plays critical roles in cancer progression, including growth, metastasis and angiogenesis. Ewing sarcoma is a sarcoma with poor prognosis despite current therapies, particularly for patients with advanced-stage disease. Lungs and bone (marrow), organs of predilection for (primary/metastatic) Ewing sarcoma, represent predominant CXCL12 sources.

**Methods:**

To gain insight into the role of the CXCR4-CXCL12 axis in Ewing sarcoma, CXCR4, CXCL12 and hypoxia-inducible factor-1α protein expression was studied in therapy-naïve and metastatic tumors by immunohistochemistry. CXCR4 function was assessed *in vitro*, by flow cytometry and proliferation/ cell viability assays, in the presence of recombinant CXCL12 and/or CXCR4-antagonist AMD3100 or under hypoxic conditions.

**Results:**

Whereas CXCR4 was predominantly expressed by tumor cells, CXCL12 was observed in both tumor and stromal areas. Survival analysis revealed an (expression level-dependent) negative impact of CXCR4 expression (p < 0.04). A role for the CXCR4-CXCL12 axis in Ewing sarcoma growth was suggested by our observations that i) CXCR4 expression correlated positively with tumor volume at diagnosis (p = 0.013), ii) CXCL12 was present within the microenvironment of virtually all cases, iii) CXCL12 induced proliferation of CXCR4-positive Ewing sarcoma cell lines, which could be abrogated by AMD3100. CXCR4 expression was not correlated with occurrence of metastatic disease. Also, therapy-naïve tumors demonstrated higher CXCR4 expression as compared to metastases (p = 0.027). Evaluation of *in vivo* hypoxia-inducible factor-1α expression and culture of cells under hypoxic conditions revealed no role for hypoxia in CXCR4 expression.

**Conclusions:**

Together, our results imply a crucial role for the CXCR4-CXCL12 axis in auto- and/or paracrine growth stimulation. Integration of CXCR4-targeting strategies into first- and/or second-line treatment regimens may represent a promising treatment option for Ewing sarcoma.

## Background

The chemokine network, initially described as an essential mediator of directional cell migration in inflammation and immune cell homing, has become increasingly recognized as contributing to a broad spectrum of other physiological and pathological processes, including cancer [1]. Although cancers of different histological origin express different chemokine receptors and/or (corresponding) ligands, chemokine receptor CXCR4 together with its cognate ligand CXCL12 (stromal cell-derived factor-1/SDF-1) is the most widely expressed (as reviewed by [2]). Constitutive CXCR4 expression has been detected in a range of adult tissues, including hematopoietic cells, vascular smooth muscle and endothelial cells and epithelial cells of different origin, whereas CXCL12 is constitutively expressed by stromal cells within the lungs and bone marrow microenvironment [2]. Hypoxia-inducible factor-1α (HIF-1α), a well-characterized inducer of gene transcription in hypoxic cells, induces expression of both CXCL12 and CXCR4 in ischemic areas [3,4]. Physiologically, the CXCR4-CXCL12 axis has important roles in hematopoiesis, development and organization of the immune system and (ischemic) tissue repair and regeneration. In cancer, this axis has been reported to play critical roles in tumor progression, including promotion of tumor cell proliferation and survival [5], metastatic processes [6] and angiogenesis [7]. Currently, after having demonstrated anti-tumor activity in pre-clinical and animal tumor models [8], several CXCR4 antagonists are being evaluated in clinical studies for treatment of patients with hematological and solid tumors [9].

Ewing sarcoma (EWS) is an aggressive round cell sarcoma affecting bone or, rarely, soft tissue in predominantly children and young adults [10]. This tumor is characterized by specific gene fusions most commonly containing *TET* gene family products, and rarely other activating transcription factors [11,12]. Despite current multimodal therapies, survival of patients has not improved significantly during the past decade. Patients with refractory and/or (primary) metastatic disease have the most unfavorable prognosis, which has recently been demonstrated to be independent of gene fusion type [13,14]. Organs of predilection for EWS metastases are lungs and bone (marrow), which represent rich sources of CXCL12. Recently, high *CXCR4* gene expression was reported to associate with metastatic phenotype in EWS [15]. Moreover, CXCL12 has been demonstrated to contribute to neovascularization and EWS tumor growth in a mouse xenograft model [16]. As yet, no information exists on CXCR4/CXCL12 protein expression and their (functional) consequences in EWS.

To gain insight into the role of the CXCR4-CXCL12 axis in EWS biology, CXCR4 expression and functionality (in the presence of CXCL12 and/or CXCR4-antagonist AMD3100) were evaluated in a large panel of therapy-naïve and metastatic tumors and cell lines, respectively. We demonstrate an expression level-dependent negative impact of CXCR4 protein expression on patients’ overall survival and point to a crucial role for auto- and/or paracrine growth signaling via the CXCR4-CXCL12 axis.

## Methods

### Ewing sarcoma patients and samples

Formalin-fixed, paraffin-embedded therapy-naïve (n = 44) as well as (sequential) metastatic (n = 16) EWS samples from 47 different patients were retrieved from the Department of Pathology, LUMC and a tissue array containing 2mm-diameter tissue-cores (Institute of Pathology, Heinrich-Heine University, Dusseldorf, Germany). Histology and tumor content were verified by a specialized bone pathologist (PCWH). Diagnosis was established according to WHO criteria, including standard confirmatory immunohistochemistry and fusion transcript type. For patients with clinical information available (Additional file 1: Table S1), mean age at diagnosis was 19 years (range 1–43 years). Follow-up (mean/median duration of follow-up: 60/44 months, respectively) provided information concerning (initial) disease extension, chemotherapy response, recurrence rate and performance state. All patient material was coded, such that code breaking and correlation with clinical data were only possible for physicians involved in treatment of the patients. Subsequent research was conducted following the ethical guidelines of the national organization of scientific societies (FEDERA).

### Ewing sarcoma cell lines

EWS cell lines EW3, RD-ES, SK-ES-1, SK-N-MC, CADO-ES and STA-ET2.1 [17] and breast cancer cell line MCF-7 (ATCC, Rockville, MD) were cultured in RPMI-1640 supplemented with streptomycin/ penicillin (Invitrogen, Paisley, United Kingdom) and 10% fetal bovine serum ((FBS); Greiner Bio-One, Alphen a/d Rijn, The Netherlands). TC71 [17] and IOR/BER (kindly provided by dr. K. Scotlandi, Instituto Orthopedico Rizzoli, Bologna, Italy) were cultured in Iscove’s Modified Dulbecco’s Medium supplemented with streptomycin/ penicillin and 10% FBS. For proliferation assays, cells (at densities ranging from 3-15x10^3^ cells/ well in 96-well-plates) were cultured for seven days in serum-free medium in the absence or presence of 100ng/ml CXCL12, 1000ng/ml AMD3100 or both. Afterwards, cell viability was measured by 3-(4,5-dimethyl-thiazol-2-yl)-5-(3-carboxymethoxy-phenyl)-2-(4-sulfophenyl)-2H-tetrazolium (MTS) cell viability assay (Promega Benelux, Leiden, The Netherlands), a colorimetric method for determining the number of viable cells in proliferation assays. The MTS tetrazolium compound is bioreduced by cells into a colored product that is soluble in tissue culture medium. The quantity of colored product as measured by the absorbance at 490nm is directly proportional to the number of living cells in culture. Submission of cells to 24-hours of hypoxia was performed in 0.1% O_2_, 5% CO_2_, balance N_2_ in a MiniGalaxy incubator (RS Biotech, Irvine, UK). The effectiveness of this approach for induction of hypoxia/ HIF-1α expression has recently been demonstrated [18]. Cell lines were routinely screened for mycoplasma contamination. Periodical authentication was performed by Short Tandem Repeat profiling and molecular HLA typing.

### Antibodies and reagents

The antibodies used for staining of antigens by immunohistochemistry and flow cytometry are described in Additional file 2: Table S2. Recombinant human CXCL12 was obtained from R&D Systems (cat.no. 350-NS/CF; Abingdon, UK). AMD3100 (octahydrochloride hydrate) was purchased from Sigma-Aldrich (A5602; Zwijndrecht, Netherlands).

### Flow cytometric analysis of CXCR4 surface expression

Flow cytometric analysis was performed on a FACScalibur (Beckton Dickinson, Franklin Lakes, NJ) and results were analyzed using Cellquest software. In short, cells were collected, centrifuged, washed in 1% BSA/ PBS, stained with primary anti-CXCR4 antibody and, subsequently, stained with a fluorochrome-labelled secondary antibody. Ligand expression was represented as fold increase in Mean Fluorescence Intensity (MFI) over isotype control staining (MFI-ratio).

### Immunohistochemistry for detection of CXCR4, CXCL12 and HIF-1 expression in Ewing sarcoma tumor samples

4μm sections containing representative tumor, as verified by a specialized bone pathologist (PCWH), were deparaffinized and citrate antigen retrieval was performed. Subsequent immunohistochemical stainings were performed and (semi-quantitatively) scored according to the quality control system as proposed by Ruiter *et al.*: the intensity of staining was scored as 0, 1, 2 or 3 indicating absent, weak, moderate or strong expression, respectively. Percentages of positive cells were scored as 0 for 0%, 1 for 1–5%, 2 for 5–25%, 3 for 25–50%, 4 for 50–75%, and 5 for 75–100%. The sum of both scores was used to identify four categories of expression: absent (0–2), weak (3–4), moderate (5–6), strong/ homogeneous (7–8) [18,19]. (Decalcified) tonsil tissue sections were used as positive control slides during initial optimization of immunohistochemical staining procedures. A decalcification procedure (formic acid) was applied to determine its influence on the immunoreactivity of epitopes. Quality of samples was guaranteed by (previous) immunohistochemical staining for CD99.

### Statistical analyses

Statistical analyses were performed with SPSS version 16.0 software package. Survival analyses were performed according to Kaplan Meier and differences in survival curves were assessed with the log-rank test. Pearson correlation analysis was used for assessment of associations between expression levels within individual samples. T-tests or repeated measures ANOVA with Bonferroni’s multiple comparison *post hoc* tests were used for comparison of expression levels between samples and associations between expression levels and clinicopathological parameters. P < 0.05 was considered statistically significant.

## Results

### CXCR4 expression in Ewing sarcoma: negative impact on survival

**Figure 1:**
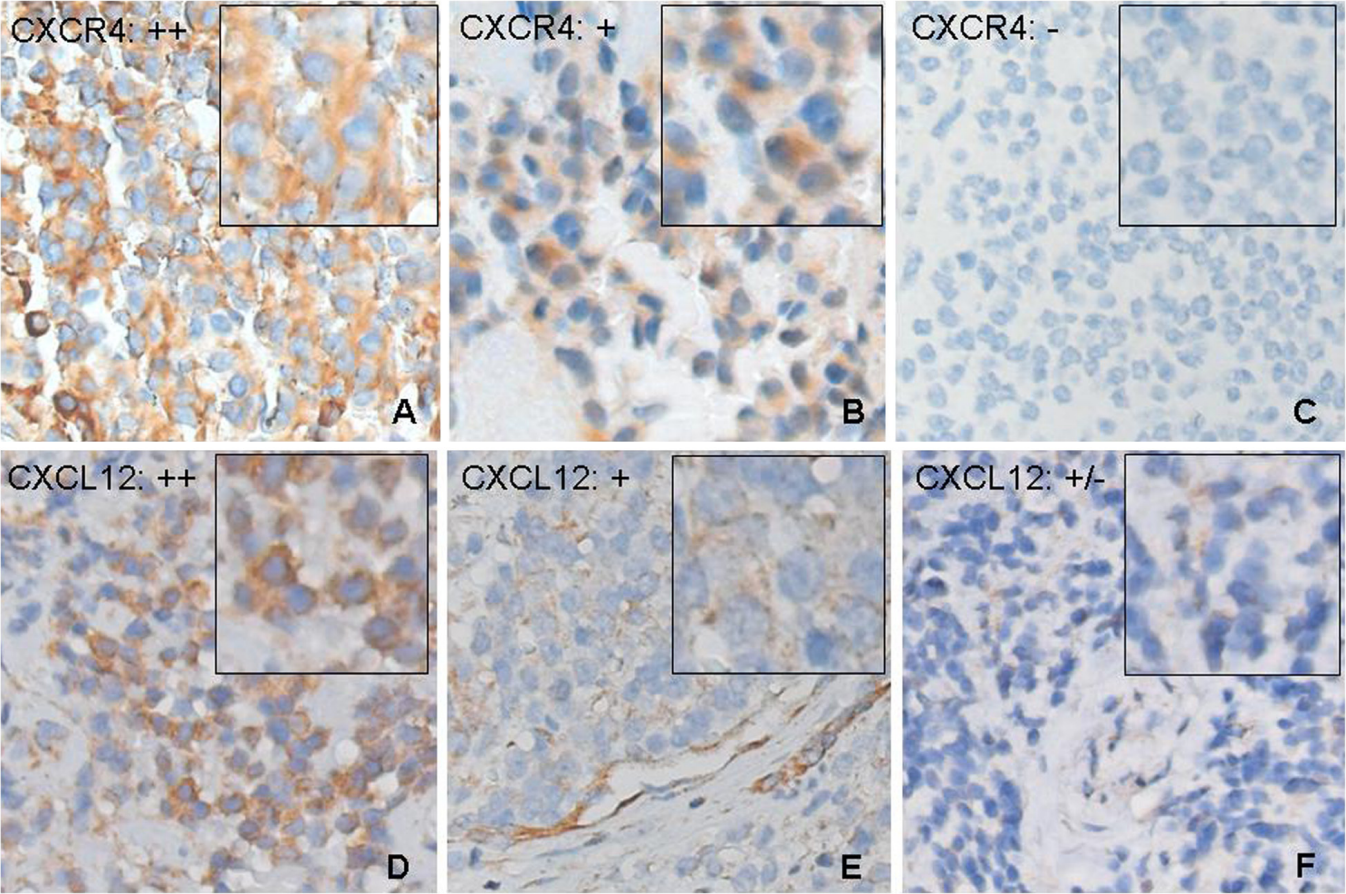
**Immunohistochemical analysis of expression of the CXCR4-CXCL12 axis in Ewing sarcoma. AF**. Light micrographs (20x magnification) of immunohistochemical stainings for CXCR4 and CXCL12 expression in therapy-naive and metastatic EWS. Representative examples. A-C: strong (++), moderate (+) and absent (−) CXCR4 expression, respectively. D-F: strong (++), moderate (+) and weak (+/−) CXCL12 expression, respectively. Whereas CXCR4 was expressed solely by tumor cells, CXCL12 was observed in both tumor and stromal areas. Insets: immunoreactivity for both CXCR4 and CXCL12 was exclusively localized in the cytoplasm of cells. Intensity of staining was (semi-quantitatively) scored according to the quality control system as proposed by Ruiter *et al.* (see Methods section) [19]. (Decalcified) tonsil tissue sections were used for initial optimization of immunohistochemical staining procedures.

**Figure 2:**
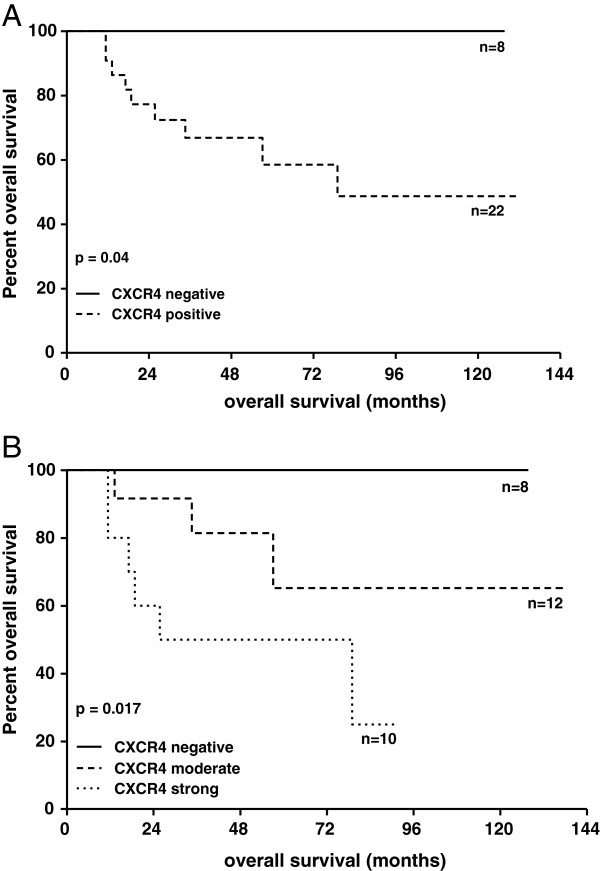
**Negative impact of CXCR4 expression in therapy-naïve Ewing sarcoma.** Kaplan-Meier survival analysis. CXCR4 expression level plotted according to the cut-off values as shown in Table 1: CXCR4 negative (absent (−) expression), CXCR4 positive (weak (+/−), moderate (+) or strong (++) expression). **A.** Inferior survival of patients with CXCR4 positive therapy-naïve tumors compared to patients with tumors lacking expression of this protein. **B.** Expression level-dependent impact of CXCR4 expression on patients’ overall survival. Inferior survival of patients with therapy-naïve tumors demonstrating strong CXCR4 expression compared to patients with weak-moderate or absent expression of this protein.

A panel of therapy-naïve (n = 44) and metastatic (n = 16) EWS samples were evaluated by immunohistochemistry for CXCR4 and CXCL12 expression. Variation between as well as within individual samples was observed, ranging from complete lack of expression of CXCR4 and CXCL12 to homogeneous expression of these proteins (Table 1; Figure 1A-F). Immunoreactivity for both CXCR4 and CXCL12 was exclusively localized in the cytoplasm of cells. Whereas CXCR4 was solely expressed by tumor cells, CXCL12 was observed in both tumor and stromal areas. As demonstrated in Table 1, CXCR4 expression was observed in 64% (28/44) of therapy-naïve and 47% (7/15) of metastatic tumors. CXCL12 was detectable in 65% (28/43) of therapy-naïve and 81% (13/16) of metastatic lesions. Noteworthy, stromal CXCL12 expression was detectable in nearly all cases (in 95% (41/43) of therapy-naïve cases and in all metastatic lesions), regardless of CXCR4/ CXCL12 expression by tumor cells. No correlation existed between CXCR4 and CXCL12 expression levels within individual tumor samples (data not shown). Comparisons between sample types, however, demonstrated significantly higher CXCR4 expression levels in therapy-naïve as compared to (sequential) metastatic EWS cases (t-test, p = 0.027) (Table 1). Kaplan-Meier survival analysis revealed a negative impact of CXCR4 expression in therapy-naïve samples (n = 30) on patients’ overall survival (log-rank test, p = 0.04) (Figure 2A). Extended analyses demonstrated this impact to be expression level-dependent (log-rank test, p = 0.017) (Figure 2B). No such effect was observed for CXCL12 (data not shown). For cases with clinical information available (n ≥ 25), analysis of the relationship with established prognostic factors in EWS [13] showed a positive correlation between CXCR4 expression in therapy-naïve samples and tumor volume at diagnosis (< or > 200 ml) (Pearson Chi-square, p = 0.013). No correlations were observed with tumor site, disease extension, histologic response to chemotherapy or relapsed metastatic disease (Additional file 1: Table S1). Due to limited sample size, multivariate analysis to assess CXCR4 expression as independent prognostic factor in Ewing sarcoma could not be performed.

**Table 1:** Immunohistochemical expression analysis of the CXCR4-CXCL12 axis in Ewing sarcoma

**UPN**^**a**^	**sample type (years after diagnosis)**	**CXCR4**^**b**^	**CXCL12**	
			**tumor**	**stroma**
1	lung metastasis (3)	-	-	+/−
	bone metastasis (7)	-	-	+/−
2	lung metastasis (2)	-	+	+
3	therapy-naive biopsy	+/−	+	+
	lung metastasis (5,5)	-	-	+/−
4	therapy-naive biopsy	++	+/−	+
	lung metastasis (3)	-	+	++
	lung metastasis (4)	-	+	++
5	therapy-naive biopsy	++	+/−	++
	bone metastasis (1)	n.e.	+/−	+
6	therapy-naive biopsy	+	++	n.e.
	lung metastasis (1)	+/−	+/−	+/−
7	therapy-naive biopsy	++	++	+
8	therapy-naive biopsy	++	+/−	+
	bone metastasis (2,5)	+	++	++
9	therapy-naive biopsy	-	+	+
	lung metastasis (0,5)	+	+	++
10	therapy-naive biopsy	+	-	+/−
11	therapy-naive biopsy	-	+	+/−
12	therapy-naive biopsy	+	+/−	+
13	therapy-naive biopsy	++	-	+/−
14	therapy-naive biopsy	-	+/−	+
15	therapy-naive biopsy	-	-	+/−
16	therapy-naive biopsy	-	+	+/−
17	therapy-naive biopsy	-	+	+
18	therapy-naive biopsy	+	+/−	+/−
19	therapy-naive biopsy	-	+/−	+
20	therapy-naive biopsy	+/−	-	+
21	therapy-naive biopsy	-	-	+/−
22	therapy-naive biopsy	-	-	-
23	therapy-naive biopsy	-	+/−	+/−
24	therapy-naive biopsy	+/−	+/−	+/−
25	therapy-naive biopsy	++	-	-
26	therapy-naive biopsy	-	+	+
27	therapy-naive biopsy	+	+/−	+
28	therapy-naive biopsy	++	+/−	+
29	therapy naive biopsy	-	-	+/−
30	therapy-naive biopsy	++	++	+
31	therapy-naive biopsy	+	-	++
32	therapy-naive biopsy	+	+	+
33	therapy-naive biopsy	-	-	+/−
34	therapy-naive biopsy	++	+	+
35	therapy-naive biopsy	+	-	+/−
36	therapy-naive biopsy	+	+	+
37	therapy-naive biopsy	+	-	+
38	therapy-naive biopsy	++	n.e.	n.e.
39	therapy-naive biopsy	++	-	+/−
40	therapy-naive biopsy	-	-	+/−
41	therapy-naive biopsy	+	+	+
42	therapy-naive biopsy	+	-	+/−
43	therapy-naive biopsy	-	++	+
44	therapy-naive biopsy	-	+	+
45	lung metastasis (1,5)	+/−	+/−	++
	bone metastasis (2,5)	++	+/−	+
	lung metastasis (2,5)	-	+	++
	lung metastasis (4)	-	+/−	++
	lung metastasis (4)	+/−	+/−	+
	lung metastasis (5)	+	+	+
46	therapy-naive biopsy	+	+	+
47	therapy-naive biopsy	+/−	++	++

^a^UPN = unique patient number. n.e. = not evaluable; - = absent expression; ‘+/− = weak expression; + = moderate expression; ++ = strong expression. ^b^t-test: therapy-naive Ewing sarcoma demonstrated significantly higher CXCR4 expression levels as compared to metastatic lesions (p = 0.027).

### A crucial role for the CXCR4-CXCL12 axis in promotion of Ewing sarcoma growth

The observed correlation between CXCR4 expression and tumor volume might either be a reflection of decreased oxygen concentrations in larger/ fast-growing tumors resulting in HIF-1α-induced CXCR4 expression, or might be caused by increased tumor cell proliferation via auto- and/or paracrine CXCL12/ CXCR4-mediated growth signalling. To explore the first potential mechanism, hypoxia-induced CXCR4 expression and the correlation between CXCR4 and HIF-1α protein expression levels were studied in EWS cell lines (n = 8) cultured under suboptimal conditions and therapy-naïve tumors (n = 38), respectively.

**Figure 3:**
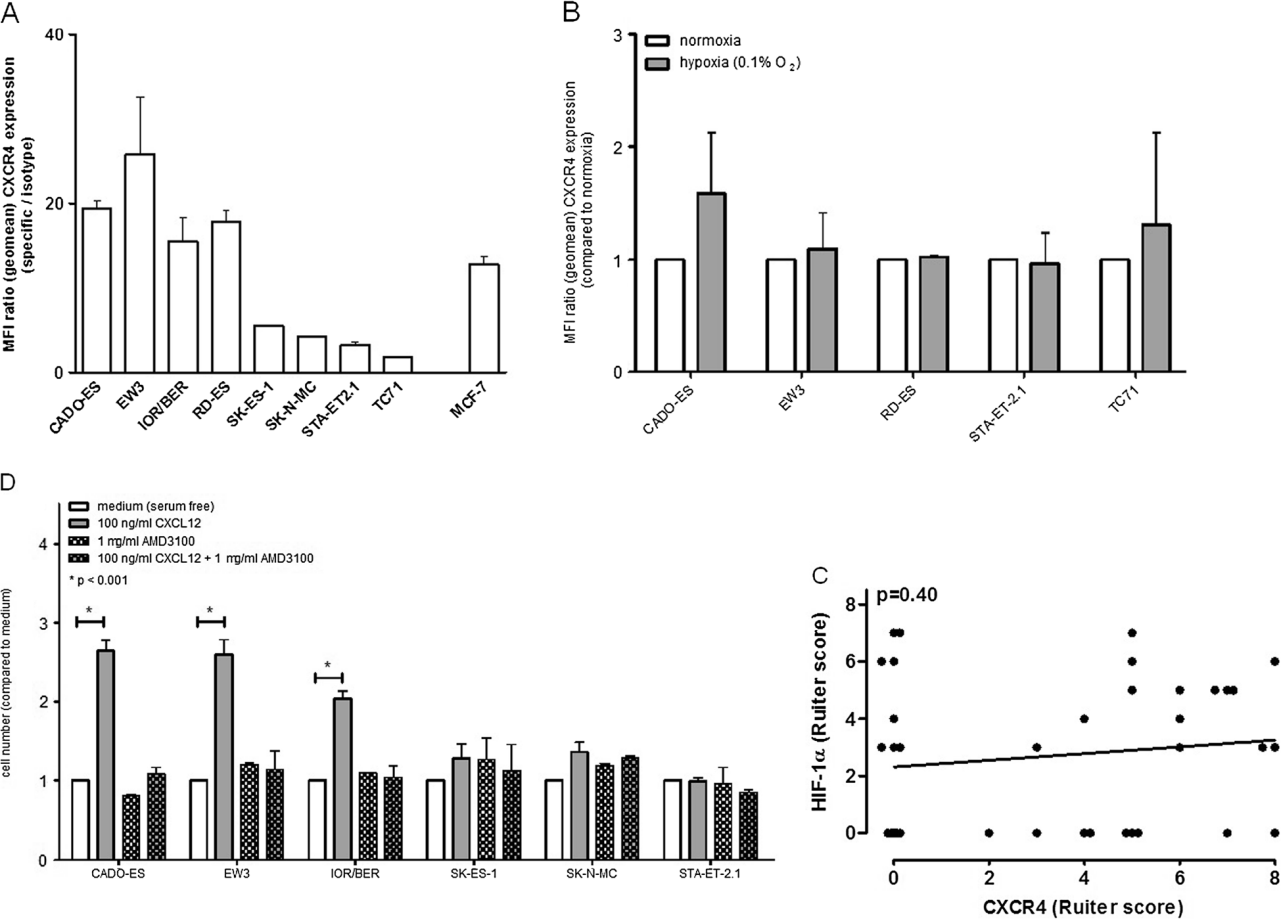
**Expression and functionality of CXCR4 in Ewing sarcoma: role for CXCL12-dependent modulation of tumor cell proliferation. A**. Constitutive surface expression of CXCR4 in EWS cell lines, as assessed by flow cytometry. Results are expressed as the mean ± SD MFI-ratio, obtained in at least two independent experiments. Breast cancer cell line MCF-7 was used as a positive control. **B.** Flow cytometric analysis of 24-hours hypoxia (0.1% O_2_)-induced CXCR4 expression in cell lines having either substantial (CADO-ES, EW3, RD-ES) or minimal (STA-ET2.1, TC71) levels of constitutive CXCR4 expression. Results are expressed as the mean ± SD fold increase in MFI-ratio over normoxic control, obtained in at least two independent experiments. Culture under hypoxic conditions (as previously described [18]) did not systematically affect CXCR4 expression. **C.** Pearson correlation analysis: lack of correlation between *in vivo* HIF-1α [18] and CXCR4 protein expression levels in 38 therapy-naïve EWS. **D.** Stimulation of cell lines expressing substantial levels of CXCR4 (CADO-ES, EW3, IOR/BER) with 100ng/ml recombinant CXCL12 for seven days significantly increased cell numbers. Addition of AMD3100 (1μg/ml) abrogated the increase in cell numbers. No effects were observed in cell lines with minimal levels of CXCR4 expression (SK-ES-1, SK-N-MC, STA-ET2.1), nor did AMD3100 treatment alone affect cell proliferation. Cells were cultured in serum-free medium. Cell viability was measured by MTS cell viability assay, a colorimetric method for determining the number of viable cells in proliferation assays (see Methods section). Results are expressed as the mean ± SD fold increase in cell numbers over (untreated) medium control, obtained in at least two independent experiments.

Whereas flow cytometric analysis revealed detectable constitutive CXCR4 surface expression in all eight cell lines evaluated, only four cell lines (CADO-ES, EW3, IOR/BER, RD-ES) demonstrated substantial levels of expression of this protein (Mean Fluorescence Intensity-ratio (over isotype control) >10), comparable to (positive control) breast cancer cell line MCF-7 (Figure 3A). Stabilization of HIF-1α protein in response to hypoxia has previously been demonstrated in EWS cell lines [18,20]. To evaluate the impact of hypoxia/ HIF-1α activation on CXCR4 expression in EWS, cell lines expressing either substantial (CADO-ES, EW3, RD-ES) or barely detectable (STA-ET2.1, TC71) levels of CXCR4 were subjected to 24-hours of hypoxia (0.1% O_2_). Culture under hypoxic conditions, compared to normoxia, did not systematically affect CXCR4 surface expression (despite successful induction of VEGF, as previously described [18]) (Figure 3B). Consistent with these findings, correlation analysis of *in vivo* HIF-1α [18] and CXCR4 expression revealed lack of correlation between expression levels of these proteins within individual tumor samples (Figure 3C).

To assess the possible contribution of the CXCR4-CXCL12 axis to EWS proliferation, cell lines were cultured in serum-free medium in the absence or presence of recombinant CXCL12. As demonstrated in Figure 3D, stimulation with 100ng/ml CXCL12 for seven days significantly increased cell numbers in cell lines expressing substantial levels of constitutive CXCR4 (CADO-ES, EW3, IOR/BER; repeated measures ANOVA with Bonferroni’s multiple comparison *post hoc* test, p < 0.001), whereas no effects were observed in cell lines with minimal levels of constitutive CXCR4 expression (SK-ES-1, SK-N-MC, STA-ET2.1). Addition of CXCR4-antagonist AMD3100 at 1μg/ml completely abrogated the CXCL12-induced proliferation of EWS cell lines. AMD3100 treatment alone did not affect (spontaneous) cell proliferation (Figure 3D).

## Discussion

Expression of the CXCR4-CXCL12 axis has been reported to coordinate events critical to tumor development and/or progression in (solid) tumors of different histological origin [2]. The present study demonstrates an (expression level-dependent) negative prognostic impact of CXCR4 protein expression in therapy-naïve EWS and points to a role for the CXCR4-CXCL12 axis in promotion of EWS cell growth. CXCL12-dependent modulation of tumor cell proliferation and survival (under suboptimal conditions) has been observed in several tumor types, including ovarian carcinoma [21], small cell lung cancer [22] and prostate cancer [23]. Here, we demonstrate positive correlations between CXCR4 expression levels in therapy-naïve EWS and tumor volume at diagnosis. Moreover, and consistent with previous gene expression results [15,24], we show expression of CXCL12 protein by most EWS tumors (65%) and, explicitly, within the tumor microenvironment of virtually all (>95%) EWS cases. Combined, these observations may reflect the existence of auto- and/ or paracrine growth stimulatory loops, mediated by the CXCR4-CXCL12 axis. Indeed, *in vitro* functional analyses demonstrate CXCL12-induced proliferation of EWS cell lines expressing substantial levels of CXCR4, which could be inhibited by CXCR4-antagonist AMD3100. Addition of AMD3100 alone did not interfere with spontaneous cell proliferation, suggesting a predominant role for paracrine (stroma-derived CXCL12) rather than autocrine (tumor cell-derived CXCL12 [24]) signalling. No correlations were observed with other established prognostic factors in Ewing sarcoma. Due to limited sample size, multivariate analysis to assess CXCR4 expression as independent prognostic factor in Ewing sarcoma could not be performed. Moreover, due to the nature of this (bone) tumor, attempts to establish primary tumor cell cultures from therapy-naive biopsies for evaluation of CXCL12-induced proliferation have so far been unsuccessful. Nuclear localization of CXCR4 has been described, and demonstrated to correlate with disease progression, in several distinct cancer types [25,26]. Within our series of therapy-naive and metastatic Ewing sarcoma, however, no nuclear accumulation of CXCR4 has been observed.

Recently, *CXCR4* gene expression was reported to associate with both EWS and osteosarcoma metastases [15,27]. Although we previously observed a correlation between *CXCR4* gene expression and disease extension/ metastatic disease at diagnosis (unpublished results), the current study does not shown any correlation between CXCR4 protein expression and occurrence of metastatic disease. Moreover, metastatic EWS lesions demonstrated significantly lower CXCR4 protein expression levels as compared to (corresponding) therapy-naïve tumors. Reduced expression of CXCR4 in metastatic lesions as compared to corresponding primary tumors has been reported in breast carcinoma, and hypothesized to be due to CXCL12-induced internalization and degradation and/or lower microenvironmental HIF-1α levels [28]. With regard to EWS, no significant differences in CXCL12 protein expression levels (in neither tumor nor stromal areas) were observed between therapy-naïve and metastatic lesions (data not shown). Moreover, although no data exist on HIF-1α expression in metastatic EWS lesions, our *in vitro* and *in vivo* analyses revealed no effect of hypoxia on CXCR4 expression nor a correlation between HIF-1α and CXCR4 expression levels (Figure 3B and Figure 3C, respectively). An alternative explanation for the observed reduced expression of CXCR4 in metastatic as compared to therapy-naïve EWS lesions might be that the CXCR4-CXCL12 axis is essential for retention of EWS cells within the primary tumor site, as has been described for CD34^+^ hematopoietic stem cells and leukemic cells within the hematopoietic microenvironment [2] and, more recently, for osteosarcoma [29]. Hypothetically, reduced expression of CXCR4 might result in preferential metastasizing of individual cells, provided that alternative growth factors are present. Whether the apparent discrepancy in correlation of *CXCR4* gene transcript ( [15]) and CXCR4 protein expression (current study) with metastatic disease in EWS reflects true biological differences (e.g. differences at the mRNA level are not reflected at the protein level (or *vice versa*), due to post-transcriptional and/or -translational regulation) or are attributable to technical differences (e.g. different samples and/or sensitivity and dynamic ranges of the methods used for mRNA transcript and protein analysis) is not known. Based on our results, we delineate a role for the CXCR4-CXCL12 axis in promotion of EWS cell growth rather than its metastatic potential.

Hypoxia is a common phenomenon in (large and/or fast-growing) solid tumors, which is associated with therapy-resistance and represents an independent prognostic indicator of poor outcome. HIF-1α, being the best characterized inducer of gene transcription in hypoxic cells, is overexpressed in various cancer types including EWS [18,20,30], and a key role for this protein in hypoxic induction of CXCR4 has been described [3,31]. Although the observed positive correlation between CXCR4 expression in therapy-naïve EWS and tumor volume at diagnosis might have been indicative for hypoxia-induced HIF1α-dependent CXCR4 activation, our analyses did not provide support for a contribution of hypoxia to CXCR4 expression in this tumor. In addition to the observed lack of correlation between HIF-1α and CXCR4 protein expression within individual tumor samples, culture of cell lines under hypoxic conditions did not affect CXCR4 surface expression. These observations are in line with results previously obtained by Aryee et al., demonstrating a lack of change in CXCR4 pathway genes upon hypoxic exposure [20].

Until rather recently, CXCR4 and CXCL12 were considered exclusive partners. However, a second CXCL12-binding chemokine receptor, CXCR7, was identified and demonstrated to be involved in progression of several cancer types, including (pediatric) sarcomas [29,32,33]. In Ewing sarcoma, *CXCR7* gene expression was recently reported to associate with patient survival [15]. As yet, no data exist on CXCR7 protein expression in Ewing sarcoma. Our preliminary results point to limited expression of this chemokine receptor in Ewing sarcoma cell lines (CXCR7 surface expression, as assessed by flow cytometry, in 1/10 cell lines). *In vivo s*tudies, using Ewing sarcoma samples or either murine or human (xenografted) tumor models may provide further insight into the role of the CXCR7-CXCR4-CXCL12 axis in Ewing sarcoma.

Hitherto, our results indicate that the CXCR4/CXCL12 axis is frequently expressed in EWS and affects tumor progression and patient survival by promoting cell growth. Successful inhibition of EWS proliferation by AMD3100, one of several CXCR4-specific antagonists that are currently being evaluated for treatment of patients with both hematological and solid tumors [9] indicates that disruption of the CXCR4-CXCL12 axis may indeed interfere with EWS progression. Integration of strategies that target CXCR4 signaling into either first- or second-line treatment regimens may represent a promising treatment option for patients with EWS.

## Conclusions

Patients with Ewing sarcoma (EWS) have a poor prognosis, despite current multimodal therapy. Integration of targeted strategies into first-line treatment regimens or introduction of these approaches as second-line therapy may represent promising treatment options. Chemokine receptor CXCR4, together with its ligand CXCL12, plays critical roles in cancer progression, including growth, metastasis and angiogenesis. Lungs and bone (marrow), organs of predilection for (primary/ metastatic) EWS, represent predominant CXCL12 sources Currently, after having demonstrated anti-tumor activity in pre-clinical and animal tumor models, several CXCR4 antagonists are being evaluated in clinical studies for treatment of patients with hematological and solid tumors. Here, we demonstrate an expression level-dependent negative impact of CXCR4 protein expression on EWS patients’ overall survival and provide evidence for a crucial role for the CXCR4-CXCL12 axis in promotion of EWS cell growth. Successful inhibition of EWS proliferation by CXCR4 antagonist AMD3100 indicates that disruption of the CXCR4-CXCL12 axis may indeed interfere with disease progression and provides a rationale for integration of CXCR4-targeting strategies in first- and/or second-line treatment regimens for EWS.

## Competing interests

The authors declare that they have no competing interests.

## Authors’ contributions

All authors contributed to conception and/or design of the study. DB, SJS, SS and HJK conducted experiments and performed data analyses. DB, MWS, SS, JV, PCWH and ACL were involved in interpretation of data. All authors were involved in drafting and/or critical revision of the manuscript and read and approved the final manuscript.

## Supplementary Material

Additional file 1 **Table S1 (.xls).** Clinical and tumor characteristics for Ewing sarcoma patients with clinical information available. Click here for file

Additional file 2: **Table S2 (.xls).** Antibodies used for immunohistochemistry and flow cytometry. Click here for file
